# Correcting *versus* resolving respiratory motion in free-breathing whole-heart MRA: a comparison in patients with thoracic aortic disease

**DOI:** 10.1186/s41747-019-0107-4

**Published:** 2019-07-31

**Authors:** Robert E. Stroud, Davide Piccini, U. Joseph Schoepf, John Heerfordt, Jérôme Yerly, Lorenzo Di Sopra, Jonathan D. Rollins, Andreas M. Fischer, Pal Suranyi, Akos Varga-Szemes

**Affiliations:** 1 Division of Cardiovascular Imaging, Department of Radiology and Radiological Science, Medical University of South Carolina, 25 Courtenay Dr, Charleston, SC 29425 USA; 2 Department of Diagnostic and Interventional Radiology, Lausanne University Hospital and University of Lausanne, Rue de Bugnon 46, BH 7.84, 1010 Lausanne, Switzerland; 3 Advanced Clinical Imaging Technology, Siemens Healthcare AG, EPFL QI-E, 1015 Lausanne, Switzerland; 4 Center for Biomedical Imaging (CIBM), Rue de Bugnon 46, BH 7.84, 1010 Lausanne, Switzerland; 5 Institute of Clinical Radiology and Nuclear Medicine, University Medical Center Mannheim, Medical Faculty Mannheim, Heidelberg University, Theodor-Kutzer-Ufer 1-3, 68167 Mannheim, Germany

**Keywords:** Aorta, Dilatation, Image processing (computer–assisted), Magnetic resonance angiography, Motion

## Abstract

**Background:**

Whole-heart magnetic resonance angiography (MRA) requires sophisticated methods accounting for respiratory motion. Our purpose was to evaluate the image quality of compressed sensing-based respiratory motion-resolved three-dimensional (3D) whole-heart MRA compared with self-navigated motion-corrected whole-heart MRA in patients with known thoracic aorta dilation.

**Methods:**

Twenty-five patients were prospectively enrolled in this ethically approved study. Whole-heart 1.5-T MRA was acquired using a prototype 3D radial steady-state free-precession free-breathing sequence. The same data were reconstructed with a one-dimensional motion-correction algorithm (1D-MCA) and an extradimensional golden-angle radial sparse parallel reconstruction (XD-GRASP). Subjective image quality was scored and objective image quality was quantified (signal intensity ratio, SIR; vessel sharpness). Wilcoxon, McNemar, and paired *t* tests were used.

**Results:**

Subjective image quality was significantly higher using XD-GRASP compared to 1D-MCA (median 4.5, interquartile range 4.5–5.0 *versus* 4.0 [2.25–4.75]; *p* < 0.001), as well as signal homogeneity (3.0 [3.0–3.0] *versus* 2.0 [2.0–3.0]; *p* = 0.003), and image sharpness (3.0 [2.0–3.0] vs 2.0 [1.25–3.0]; *p* < 0.001). SIR with the 1D-MCA and XD-GRASP was 6.1 ± 3.9 *versus* 7.4 ± 2.5, respectively (*p* < 0.001); while signal homogeneity was 274.2 ± 265.0 *versus* 199.8 ± 67.2 (*p* = 0.129). XD-GRASP provided a higher vessel sharpness (45.3 ± 10.7 *versus* 40.6 ± 101, *p* = 0.025).

**Conclusions:**

XD-GRASP-based motion-resolved reconstruction of free-breathing 3D whole-heart MRA datasets provides improved image contrast, sharpness, and signal homogeneity and seems to be a promising technique that overcomes some of the limitations of motion correction or respiratory navigator gating.

## Key points

- Prototype compressed sensing whole-heart magnetic resonance angiography (MRA) provides improved signal homogeneity and sharpness.
- Motion-resolved three-dimensional whole-heart MRA reconstruction outperformed one-dimensional image-based motion correction.
- Motion-resolved MRA allows for image acquisition without the need for respiratory navigator.

## Background

Whole-heart magnetic resonance angiography (MRA) has been extensively used as a clinical tool to visualize three-dimensional (3D) cardiac anatomy [1]. While 3D whole-heart MRA can be obtained using contrast-enhanced techniques [2], the most common application remains the unenhanced steady-state free-precession (SSFP) acquisition [3–6]. As the heart is relatively still only for a short mid-diastolic or late systolic period of the cardiac cycle, collection of such MRA data requires hundreds of cardiac cycles and a protocol designed for free-breathing imaging.

Current 3D SSFP MRA techniques depend on sophisticated respiratory motion compensation or gating. Diaphragmatic navigators, the only tool clinically available, have several limitations including unpredictable acquisition time and low scan efficiency, which can render the length of the acquisition excessively long, up to 28 min [7–10]. Alternatively, a variety of self-navigated techniques have been proposed, which allow the extraction of a respiratory motion signal directly from the image data that can be used for respiratory motion correction during post-processing [11–16]. Self-navigation, employing a radial trajectory combined with one-dimensional correction, has been one of the most widely used investigational approaches which can provide 100% scan efficiency and predictable acquisition times, enabling the application of this technique even in children with limited compliance [17, 18]. However, self-navigation also suffers from certain limitations that are related to the 1D motion model used for correction [19], which may not be accurate when a wide range of respiratory motion is present [20].

Recent developments have shifted towards more sophisticated reconstruction techniques that are able to extract a respiratory signal from the image data and use it to sort images into different respiratory motion states. The extradimensional golden-angle radial sparse parallel (XD-GRASP) method, a novel compressed sensing image reconstruction framework, combines the benefits of reduced k-space sampling and sparse reconstruction [20, 21]. In particular, XD-GRASP enables the reconstruction of 3D radial golden-angle free-breathing coronary artery MRA data at multiple respiratory phases by exploiting the sparsity along the respiratory dimension [20]. This is a paradigm shift for motion compensation, as with this technique, respiratory motion is no longer corrected using an approximation of the displacement (*e.g.,* reducing the motion to a 1D correction). Instead, the strong similarity between different respiratory phases is used to improve the image quality of the reconstruction.

The purpose of this study was to evaluate the subjective and objective image quality of XD-GRASP-based respiratory motion-resolved 3D whole-heart MRA in comparison with respiratory motion-corrected whole-heart MRA in patients with known thoracic aorta dilation.

## Methods

### Patient selection

Our study protocol was approved by the local Institutional Review Board and written informed consent was obtained from all patients. The study was conducted in compliance with the Health Insurance Portability and Accountability Act guidelines. Patients (*n* = 25) with known thoracic aorta dilation were prospectively enrolled for a research study between July 2017 and September 2018. Further inclusion criteria were: (1) > 18 years of age; (2) previous clinically indicated aorta, chest, triple-rule-out, or pulmonary embolism computed tomography examination; and (3) willing to comply with all study procedures and provide written informed consent. General magnetic resonance exclusion criteria were applied to patient selection. Patient’s demographics were obtained by medical record chart review.

### Acquisition protocol

Image acquisition was performed on a 1.5-T system (Magnetom Avanto DOT, Siemens Healthcare, Erlangen, Germany). Patients were scanned head-first in a supine position. A multi-channel spine phased-array radiofrequency coil with 24 elements integrated into the patient table and a 6 element, 6-channel phased-array body coil were used for signal reception. Acquisitions were electrocardiographically gated. The entire protocol was performed in a free-breathing fashion. Following the initial scout images, a free-breathing two-dimensional balanced SSFP cine image set in a parasagittal long-axis view of the left ventricle was acquired using the following typical parameters: repetition time/echo time, 2.3/1.1 ms; field of view 220–340 mm^2^; matrix 192^2^; number of segments 15; reconstructed phases 25; temporal resolution 45 ms; flip angle 77°; number of excitations 3; and parallel acquisition acceleration factor 2. Cine image data were used to determine the optimal mid-diastolic timing for the whole-heart MRA.

Whole-heart MRA was performed using a prototype fat-saturated and T2-prepared pulse sequence that employs the 3D radial trajectory [7, 22]. A coronal saturation slab was placed at the level of the anterior chest wall and the following imaging parameters were used: repetition time/echo time 3.1/1.5 ms; field of view 320 × 320 × 320 mm^3^; reconstructed voxel size, 1.7 × 1.7 × 1.7 mm^3^; matrix 192^3^; flip angle 115°; and bandwidth, 898 Hz/pixel. An acceleration factor of 5 with respect to the Nyquist sampling for 3D radial imaging was applied [21]. The acquisition was performed in free-breathing and the following two approaches were used to address respiratory motion within the reconstruction.

### Post-processing

#### Respiratory motion-corrected approach

For the self-navigation approach, respiratory motion was extracted by cross-correlating the automatically segmented blood pool of the 1D *Fourier transform* of a readout along the superior-inferior direction acquired consistently at the beginning of each heartbeat. The detected 1D superior-inferior respiratory displacement was then used for correcting each readout before the gridding operation. The correction was performed by applying a phase shift to all k-space radial readouts and was adapted for the polar orientation of each readout according to the spiral phyllotaxis pattern [22]. This reconstruction has been implemented inline at the scanner and takes about 1–2 min. Further details about the algorithm employed for motion correction were described by Piccini et al. [12].

#### Respiratory motion-resolved approach

For the respiratory motion-resolved approach, the same raw data of the whole-heart MRA acquisition were exported and processed on a dedicated workstation using an adaptation of the previously described framework [20] implemented in MATLAB 2015a (MathWorks, Natick, Massachusetts, USA). Using a respiratory signal extracted directly from the image data, individual readouts of the 3D radial acquisition were binned according to their respiratory phase [23]. The resultant series of motion-resolved undersampled images were then reconstructed using an XD-GRASP algorithm [21], which aims at exploiting the intrinsic similarities between distinct respiratory phases (or motion states) of the whole-heart acquisition to perform a compressed sensing reconstruction along the respiratory motion dimension. To achieve this, first, the acquired readouts are separated and grouped according to the respiratory phase they belong to. Subsequently, a k-t sparse SENSE iterative reconstruction is performed, where the temporal domain is represented by the different respiratory phases. Out of the four reconstructed respiratory phases, the end-expiratory phase was selected in all datasets for the subsequent analyses. The motion resolved reconstruction has not been implemented inline and takes between 15–30 min using high-end computers.

### Image analysis

Respiratory motion-corrected and motion-resolved MRA reconstructions were randomized and independently reviewed by two readers (with 1 and 11 years of experience in cardiovascular imaging, respectively) on a dedicated workstation (Aquarius iNtuition Edition v4.4.12, TeraRecon, Inc., Foster City, CA, USA). Standard axial, coronal, and sagittal planes were used to generate multi-planar reformats (MPR), but readers were allowed to use curved MPR or maximum intensity projection (MIP) series according to their preference. The presence of artifacts was noted.

#### Qualitative analysis

The overall image quality was subjectively rated independently by each reader on a 5-point Likert-scale: (1) vascular anatomy not assessable due to severe image artifacts and/or poor contrast, (2) vascular anatomy assessable despite severe image artifacts and/or poor contrast, (3) acceptable image quality with artifacts and/or limited contrast, (4) good image quality with minor artifacts and/or good contrast, and (5) excellent image quality without artifacts and excellent contrast. Signal homogeneity in the intra-aortic blood pool was rated using a 3-point Likert-scale as (1) inhomogeneity affecting diagnosis, (2) subtle inhomogeneity with no effect on diagnosis, and (3) excellent homogeneity. Image sharpness was evaluated on a 3-point scale as follows: (1) motion affecting diagnosis, (2) motion with no effect on diagnosis, and (3) no significant motion. Finally, diagnostic confidence was also rated by each reader using a 3-point scale as (1) low reader confidence, (2) marginal reader confidence, and (3) high reader confidence.

#### Quantitative analysis

Readers visualized seven standard anatomical levels of the thoracic aorta using a double oblique technique as follows: sinuses of Valsalva, sinotubular junction, mid ascending aorta, proximal aortic arch, mid aortic arch, proximal descending aorta, and mid descending aorta [24]. At each level, the signal intensity ratio (SIR) between the intravascular signal and the surrounding lung tissue was calculated. Regions of interest were placed in the center of the aorta. Aorta blood pool signal homogeneity was quantified by measuring the standard deviation of blood signal as a function of distance along the thoracic aorta on centerline reconstructions. Finally, the sharpness of the right coronary artery was quantitatively evaluated using a dedicated prototype application (Soap-Bubble, John’s Hopkins University, Baltimore, MD, USA) [25]. This software examines a user-assisted definition of a curved subvolume enclosed in the 3D MRA dataset and measures the magnitude of local change in signal intensity at the vessel borders in the reformatted image. The resulting vessel edge value indicates quantitative sharpness, whereas a value of 100% refers to an abrupt signal intensity change at the vessel border, and lower values are associated with lower vessel sharpness [25]. Measurements were taken in the proximal segment of the right coronary artery, being the most sensitive to motion [26].

### Statistical analysis

Statistical analysis was performed using SPSS v213 (IBM Corporation, Armonk, NY, USA). Categorical variables are represented as total number and percentages, and continuous variables as mean ± standard deviation or median (interquartile range), depending on their distribution (tested with Shapiro Wilkes). Subjective image quality scores were compared between the respiratory motion-corrected and motion-resolved techniques using the Wilcoxon signed-rank test, and the McNemar test was used to compare the presence of image artifacts. Interclass correlations (ICC) were used to assess the absolute agreement between readers and were interpreted as follows: < 0.2, poor; 0.2–0.4, acceptable; 0.41–0.6, moderate; 0.61–0.8, good; and > 0.8, excellent. Objective image quality measures were compared using a paired Student’s *t* test. A *p* value < 0.05 was considered significant.

## Results

**Fig. 1:**
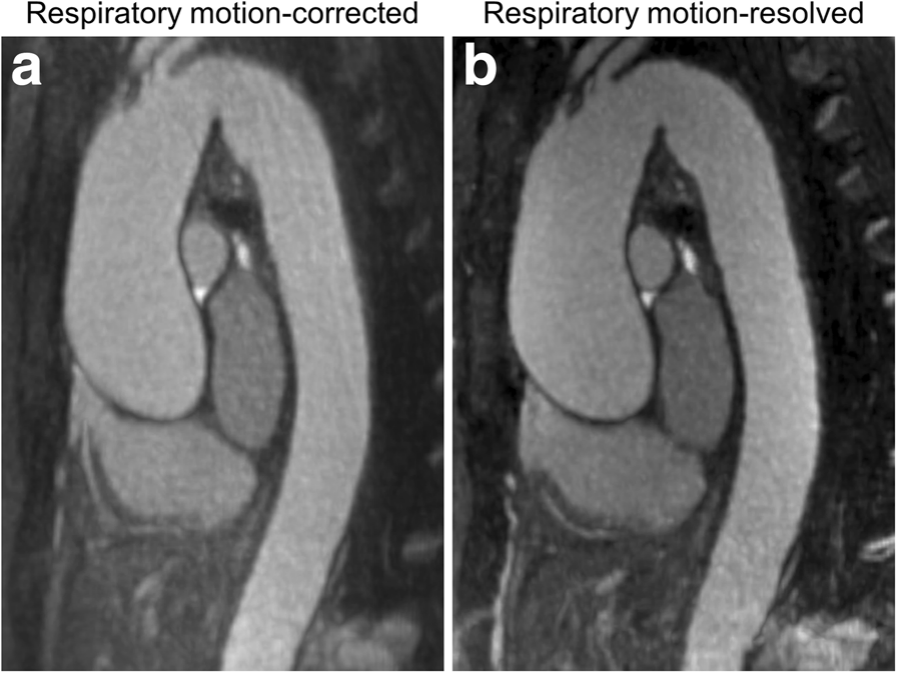
Representative images from a 66-year-old woman with ascending aorta dilation. Maximum intensity projection MRA images displayed as 3-mm thick slabs are shown in the candy cane view of the aorta reconstructed using motion-corrected (**a**) and motion-resolved (**b**) algorithms in end-expiratory phase. While the image quality of both datasets was rated the best, the improved sharpness and overall image quality achieved by the motion-resolved reconstruction can be clearly observed

A total of 25 patients with thoracic aorta dilation were enrolled, aged 70 ± 9 years (mean ± standard deviation, 16 men and 9 women). The mean patient body weight and body mass index were 88.5 ± 14.6 kg and 27.8 ± 3.0 kg/m^2^, respectively. All patients were successfully scanned using the free-breathing whole-heart MRA protocol. Representative high-quality images of respiratory motion-corrected and motion-resolved reconstructions of corresponding datasets are shown in Fig. 1.

**Fig. 2:**
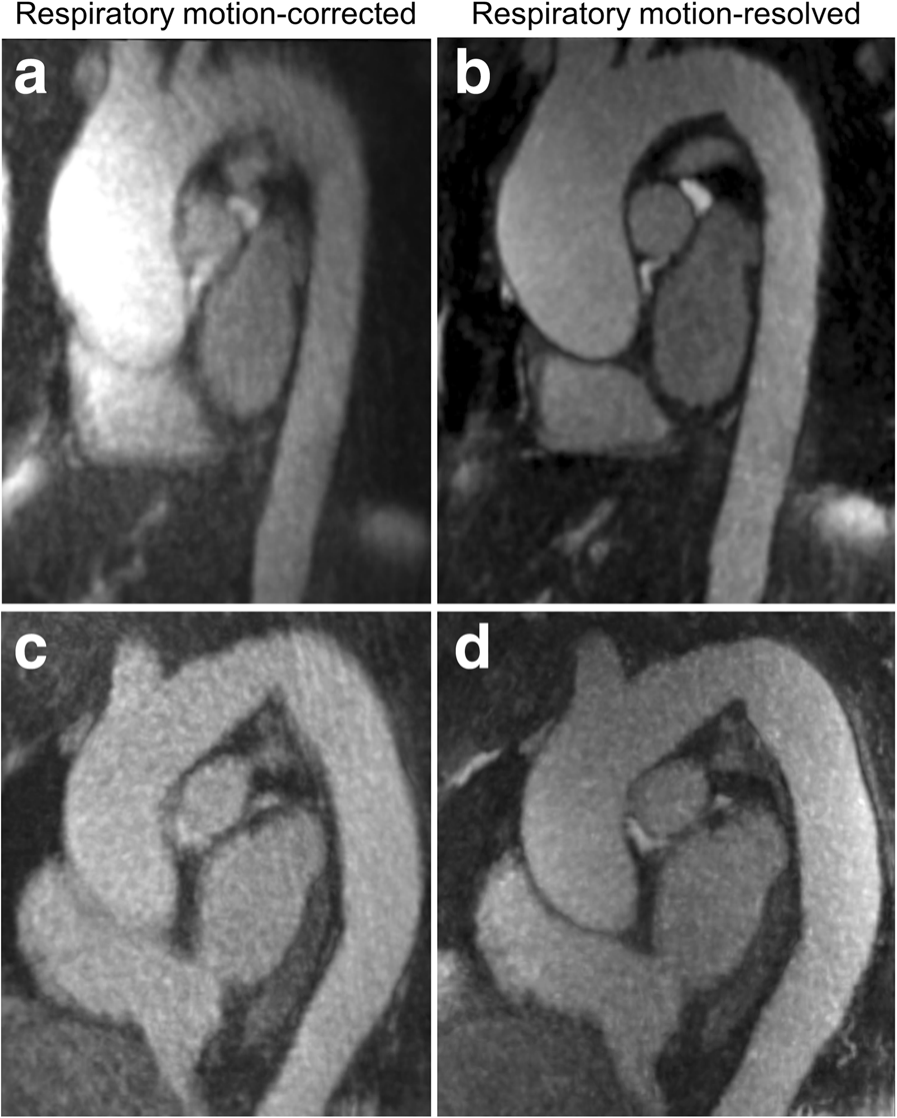
Representative motion-corrected (**a**, **c**) and motion-resolved (**b**, **d**) images from a 74-year-old woman (**a**, **b**) and a 59-year-old woman (**c**, **d**), both with ascending aorta dilation. Maximum intensity projection images displayed as 3-mm thick slabs are shown in the candy cane view of the aorta. Substantially improved image sharpness can be observed with motion-resolved reconstruction in both cases (**b**, **d**) along with improved signal uniformity especially in the first patient (**a**). Note that data from the same image acquisition are used but processed differently

Overall subjective image quality was rated significantly higher using the motion-resolved reconstruction compared to motion correction (4.5 *versus* 4.0, *p* < 0.001), including signal homogeneity and image sharpness (Table 1). Representative images highlighting the signal homogeneity and sharpness differences between the two techniques are shown in Fig. 2. Image artifacts mostly arising from motion were noted on 7 motion-corrected and 3 motion-resolved datasets (*p* = 0.219). The improved image quality of the motion-resolved reconstruction also resulted in higher diagnostic confidence scores (3.0 *versus* 2.0, *p* = 0.016). Inter-reader assessment showed moderate to excellent agreement between the readers in motion-corrected datasets (ICCs between 0.577 and 0.841) and good to excellent agreement in the motion-resolved image sets (ICCs between 0.648 and 0.860) (Table 2).

**Table 1:** Subjective image quality parameters. Data are reported as median with interquartile ranges or frequency

	Respiratory self-navigated	Respiratory motion-resolved	*p* value
Overall image quality	4.0 [2.25–4.75]	4.5 [4.5–5.0]	< 0.0001*
Signal homogeneity	2.0 [2.0–3.0]	3.0 [3.0–3.0]	0.003*
Image sharpness	2.0 [1.25–3.0]	3.0 [2.0–3.0]	0.0001*
Presence of artifacts	7 (25%)	3 (10.7%)	0.219
Diagnostic confidence	2.0 [2.0–3.0]	3.0 [2.0–3.0]	0.016*

*Indicating significant difference

**Table 2:** Inter-reader agreement in subjective image quality ratings as shown by intra-class correlation coefficient values

	Respiratory self-navigated	Respiratory motion-resolved
Overall image quality	0.841	0.860
Signal homogeneity	0.577	0.648
Image sharpness	0.784	0.772
Presence of artifacts	0.825	0.680
Diagnostic confidence	0.726	0.780

**Fig. 3:**
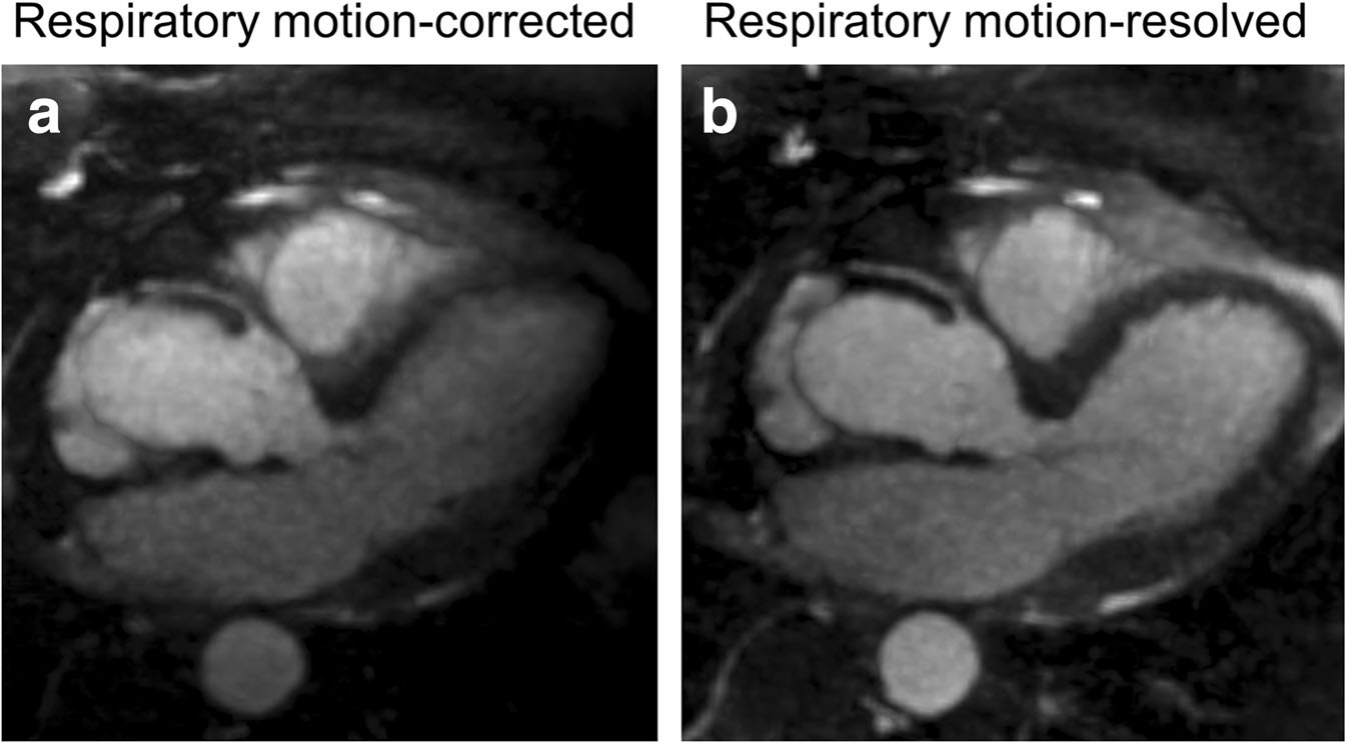
Left ventricular outflow track view of the heart visualizing the proximal segment of the right coronary artery. Motion-resolved reconstruction (**b**) provides improved sharpness (62.6%) compared to motion correction (50.9%, **a**)

Objective image quality parameters showed improvement when the motion-resolved technique was used. Overall SIR values with the motion-corrected and motion-resolved techniques were 6.1 ± 3.9 *versus* 7.4 ± 2.5 (*p* < 0.001). Individual SIR measurements taken at the various standard anatomical levels of the thoracic aorta showed significant improvement at the mid arch, proximal descending aorta, and mid descending aorta (Table 3). No statistically significant difference was observed in signal homogeneity (motion-corrected *versus* motion-resolved 274.2 ± 265.0 vs 199.8 ± 67.2, *p* = 0.129); however, the standard deviations for the motion-corrected acquisitions were higher when compared with the motion-resolved technique, indicating a more uniform signal measurement in the latter. Soap-Bubble-based image sharpness analysis revealed that motion-resolved reconstruction provides higher coronary vessel sharpness (45.3 ± 10.7 *versus* 50.6 ± 10.1, *p* = 0.025), as shown in the image example in Fig. 3.

**Table 3:** Objective image quality parameters

	Respiratory self-navigated	Respiratory motion-resolved	*p* value
Signal intensity ratio			
Sinus	6.9 ± 4.5	7.7 ± 2.2	0.325
Sinotubular junction	7.2 ± 5.4	7.8 ± 2.2	0.601
Ascending aorta	7.1 ± 5.2	7.3 ± 2.5	0.829
Proximal arch	5.6 ± 3.4	6.1 ± 1.8	0.475
Mid arch	4.7 ± 1.9	5.6 ± 1.7	0.035*
Proximal descending aorta	5.0 ± 1.3	8.0 ± 2.7	< 0.0001*
Mid-descending aorta	6.0 ± 2.8	9.2 ± 2.9	0.0001*
Signal homogeneity	274.2 ± 265.0	199.8 ± 67.2	0.129
Right coronary artery sharpness	45.3 ± 10.7	50.6 ± 10.1	0.025*

*Indicating significant difference

## Discussion

This study aimed to evaluate if respiratory-resolved compressed sensing reconstruction, specifically XD-GRASP, provides improved image quality of free-breathing 3D whole-heart MRA, when compared to self-navigated motion-corrected reconstructions of the same sets of data in a patient population. Subjective and objective image quality measures were assessed by two readers. Overall, we found improved image quality and better diagnostic confidence using the respiratory motion-resolved reconstruction, indicating that the technique has the potential to overcome the limitations of other approaches used for respiratory motion compensation or correction.

In this study, we reported improvements in both subjective and objective image quality parameters when the motion-resolved technique was used. Subjective ratings were higher in the overall assessment, but also showed a significant increase when signal homogeneity and image sharpness were rated separately. The improvement in image quality also resulted in an increase in the readers’ diagnostic confidence. Inter-reader assessment showed similar agreement between the readers for both motion-corrected and motion-resolved.

Vascular signal in the motion-corrected data sets proved to be less uniform, especially in the center of the image, most likely because the compressed sensing reconstruction technique reduced the standard deviation of the noise. Such inhomogeneity was not observed on motion-resolved reconstructions, despite the utilization of the same raw data for both algorithms. Although the quantitative signal homogeneity assessment did not show any significant difference, a substantially higher standard deviation can be observed in the motion-corrected datasets, which potentially contributed to the statistical outcome.

Coronary vessel sharpness, on the other hand, was also found to be significantly improved when the respiratory motion-resolved reconstruction was used. The Soap-Bubble analysis confirmed the increase of sharpness, which otherwise can also be visually observed. The quantitative vessel edge sharpness values measured in the motion-resolved datasets in this study population were comparable to those obtained using respiratory navigator-gated MRA [25] and higher than those reported with self-navigated motion-corrected whole-heart MRA with or without contrast administration [7, 17]. A previous study performed in a limited number of healthy volunteers reported similar improvement in vessel edge sharpness using the motion-resolved reconstruction when compared to motion correction [20]. Our study, however, was different in multiple aspects. We not only set out to use the technique in a cohort of clinical patients, creating a more relevant study design, but we also imaged the entire thoracic aorta. The latter required the use of an increased field of view (in the 300–350-mm range), which subsequently reduced spatial resolution. Despite the decrease in spatial resolution, we were able to demonstrate the superiority of the motion-resolved technique over the motion-corrected reconstruction in objective, quantifiable vessel edge sharpness. While vessel sharpness does not necessarily represent the quality of respiratory motion compensation or correction, this measure may also be influenced by other factors including cardiac motion (*e.g.,* patients with high heart rate variability) and patient movements. However, the very same datasets were used and compared in our study, meaning that the cardiac motion and patient movements were exactly the same in both reconstructions.

Finally, SIR measurements indicated significant improvement in image contrast using the respiratory motion-resolved reconstruction. The difference in SIR was the most apparent at the mid arch, proximal descending aorta, and mid descending aorta levels. One possible explanation is that the XD-GRASP reconstruction provides both a better suppression of the motion artifacts (resolving motion *versus* correcting) and a reduced amount of noise (regularization term in the reconstruction) not only in the heart and vessels, but also in the lung tissue. Since the region of interest selected within the pulmonary tissue averages pixels with relatively very low intensities, even small changes in the noise levels will produce improved SIRs.

The XD-GRASP-based reconstruction is an algorithm which outputs a motion resolved 3D whole-heart dataset without the need for any navigator or motion correction. As the reconstruction can be performed offline after the acquisition, theoretically any free-breathing radial 3D whole-heart dataset can be reprocessed without the need to acquire new data. The motion-resolved algorithm overcomes most of the limitations that diaphragmatic navigator gating or self-navigation combined with motion correction is subject to [20], such as long and unpredictable image acquisition times [27–31] or artifacts and residual motion issues with the 1D self-navigation [6, 20]. While several other groups have proposed continuous data acquisition regardless of the phase of the respiratory cycle [11, 12, 14–16], most of the techniques apply retrospective motion correction using registration algorithms or motion models [13, 14, 32, 33], which, in contrast, are not used in the XD-GRASP algorithm. The novelty in applying a respiratory motion-resolved algorithm is that the image data can be continuously acquired in a free-breathing fashion over a certain number of predetermined heartbeats, without the need for any real-time navigation or motion correction [20]. The reconstruction algorithm considers respiratory motion as an additional dimension without imposing a specific motion model for the reconstruction and allows the reader to choose the most optimal phase from the respiratory domain during the post-processing steps. Such XD-GRASP-based motion-resolved reconstruction approaches are now moving towards five-dimensional continuous imaging, where the same concept is applied at the same time to respiratory and cardiac motion [34].

Our study has some limitations to consider. While our sample size is limited, such image quality assessment does not require a large patient cohort. Our study did not demonstrate how image quality improvement affects diagnostic value; however, going forward, we are planning to expand the assessment to other aspects of the evaluation (diagnostic accuracy, coronary artery visualization, etc.) in a wider range of patients (including various age, as children with low compliance) and for different disease groups (*e.g.,* congenital heart disease). Another limitation that highly depends on the available infrastructure is the non-negligible computational power that the motion-resolved reconstruction requires. Currently, the processing time is in the 15–30-min range using high-end computers, which is expected to decrease with the continuous improvement in computer technologies. Finally, MPRs and MIPs generated individually by the two readers may have influenced visualization and consequently image quality assessment.

In conclusion, XD-GRASP-based motion resolved reconstruction of free-breathing 3D whole-heart MRA datasets provides improved image contrast, sharpness, and signal homogeneity and seems to be a promising technique that overcomes some of the limitations of motion correction or respiratory navigator gating.

## Abbreviations

1D: One-dimensional
2D: Two-dimensional
3D: Three-dimensional
ICC: Interclass correlation
MIP: Maximum intensity projection
MPR: Multi-planar reformat
MRA: Magnetic resonance angiography
SIR: Signal intensity ratio
SSFP: Steady-state free-precession
XD-GRASP: Extradimensional golden-angle radial sparse parallel
